# Extensive introgressive hybridization within the northern oriole group (Genus *Icterus*) revealed by three-species isolation with migration analysis

**DOI:** 10.1002/ece3.365

**Published:** 2012-08-29

**Authors:** Frode Jacobsen, Kevin E Omland

**Affiliations:** Department of Biological Sciences, University of Maryland Baltimore County1000 Hilltop Circle, Baltimore, MD, 21250, USA

**Keywords:** Allele sharing, coalescent, *Icterus*, IMa2, incomplete lineage sorting, introgression, multipopulation, northern orioles

## Abstract

Until recently, studies of divergence and gene flow among closely-related taxa were generally limited to pairs of sister taxa. However, organisms frequently exchange genes with other non-sister taxa. The “northern oriole” group within genus *Icterus* exemplifies this problem. This group involves the extensively studied hybrid zone between Baltimore oriole (*Icterus galbula*) and Bullock's oriole (*I. bullockii*), an alleged hybrid zone between *I. bullockii* and black-backed oriole (*I. abeillei*), and likely mtDNA introgression between *I. galbula* and *I. abeillei*. Here, we examine the divergence population genetics of the entire northern oriole group using a multipopulation Isolation-with-Migration (IM) model. In accordance with Haldane's rule, nuclear loci introgress extensively beyond the *I. galbula–I. bullockii* hybrid zone, while mtDNA does not. We found no evidence of introgression between *I. bullockii* and *I. abeillei* or between *I. galbula* and *I. abeillei* when all three species were analyzed together in a three-population model. However, traditional pairwise analysis suggested some nuclear introgression from *I. abeillei* into *I. galbula*, probably reflecting genetic contributions from *I. bullockii* unaccounted for in a two-population model. Thus, only by including all members of this group in the analysis was it possible to rigorously estimate the level of gene flow among these three closely related species.

## Introduction

The study of phylogeography and population divergence is challenged by the difficulty of distinguishing between shared retained ancestral polymorphism and introgressed alleles (Nielsen and Wakeley 2001). The much larger effective population size of nuclear DNA relative to mtDNA slows the rate of lineage sorting and the speed at which reciprocal monophyly is achieved at the nuclear level (Avise 2004). Slow and stochastic lineage sorting thus causes a pattern of allele sharing among species that can seem similar to patterns caused by gene flow through introgressive hybridization, even between long-divergent lineages (Hudson and Turelli 2003). Multiple unlinked loci and coalescent-based “divergence population genetics” methods are needed to distinguish between these two processes (e.g., Kliman et al. 2000; Machado et al. 2002).

The Isolation-with-Migration (IM) model (Hey and Nielsen 2004, 2007) provides a powerful tool for the study of population divergence and for testing different speciation models and demographic scenarios (Hey 2006; Peters et al. 2007; Kondo et al. 2008; Pinho and Hey 2010). Shared alleles allow multilocus coalescent approaches such as IM to reconstruct the divergence history of closely related species (Nielsen and Wakeley 2001; Hey and Nielsen 2004, 2007). The original IM model was designed for two-population studies between sister taxa that have not exchanged genes with other taxa since they split (Hey and Nielsen 2004, 2007). However, organisms frequently exchange genes with other non-sister taxa (Arnold 2006; Nosil 2008; Petit and Excoffier 2009). Genetic contributions from other source populations not included in the analysis (i.e., “ghost” populations) could thus severely bias or invalidate inference about the history of closely related taxa (Beerli 2004; Slatkin 2005; Strasburg and Rieseberg 2010). Thus, until recently, IM analyses were limited to pairs of taxa that share more recent common ancestry and gene flow with each other. However, this limitation was removed with the release of IMa2, allowing the simultaneous consideration of up to ten populations granted a population tree (i.e., phylogeny) is available for the taxa involved (IMa2: Hey 2010b).

**Figure 1:**
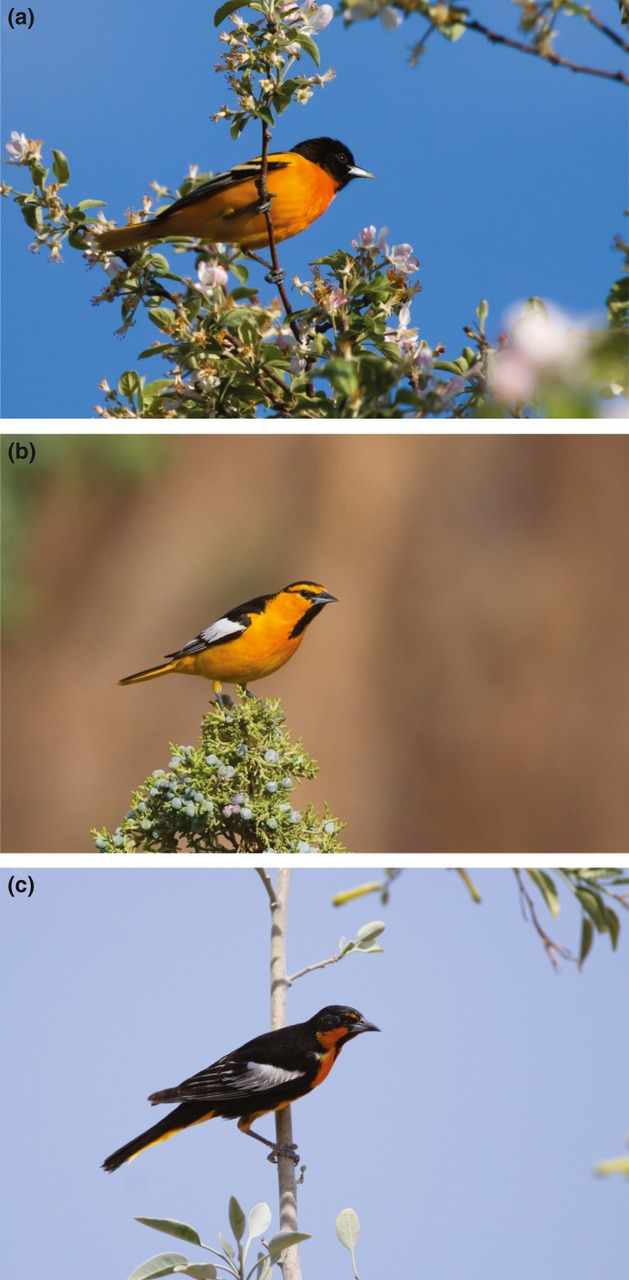
Adult males of the three species in the northern oriole group; (a) Baltimore oriole *Icterus galbula* (photo: Frode Jacobsen), (b) Bullock's oriole *I. bullockii* (photo: Frode Jacobsen), and (c) black-backed oriole *I. abeillei* (photo: Jonathan Hiley)

**Figure 2:**
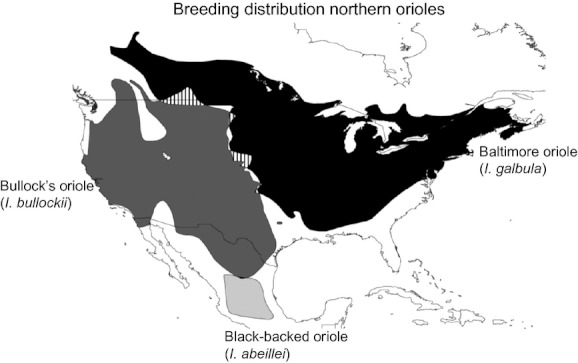
Breeding distributions of the three northern oriole species: *Icterus abeillei* (light gray), *I. bullockii* (dark gray), *and I. galbula* (black). Hatched areas indicate range overlap between *I. bullockii* and *I. galbula*. Digital distribution maps for each species were downloaded from NatureServe (Ridgely et al. 2007).

In this study, we used IMa2 to analyze the three species in the “northern oriole” group within the New World orioles (Jacobsen and Omland 2011; Fig. 1). This group includes the well-known hybrid zone between the eastern Baltimore oriole (*Icterus galbula*) and Bullock's oriole (*I. bullockii*) (Sutton 1938, 1968; Sibley and Short 1964; Rising 1969, 1970, 1983, 1996; Corbin et al. 1979). *I. galbula* breeds across the eastern United States south to Louisiana, and across most of Canada from Alberta in the west to Nova Scotia in the east (Fig. 2). The breeding range of *I. bullockii* extends across western North America from British Columbia, Canada in the north to Durango, Mexico in the south. Both *I. galbula* and *I. bullockii* are long-distance migrants and mainly overwinter in southern Mexico and Central America (Rising et al. 1998, 1999). Their winter ranges overlap with the range of the third member of the group, *I. abeillei*, a Mexican endemic limited to the central Mexican plateau (Jaramillo and Burke 1999) and traditionally considered a close relative and subspecies within *I. bullockii* due to their overall phenotypic similarity (Jaramillo and Burke 1999).

The parapatric breeding ranges of *I. galbula* and *I. bullockii* overlap along a long and narrow transect (150 km in Kansas, Allen 2002) that stretches over 1800 km from southern Alberta to central Texas (Rising 1983). The two orioles interbreed extensively in this contact zone, which falls within the “Rocky Mountain-Great Plains” suture zone (Remington et al. 1968), a hotspot of avian hybrid zones between eastern and western species pairs (e.g., Carling and Brumfield 2008; Flockhart and Wiebe 2009; Mettler and Spellman 2009). Documentation of viable hybrid offspring within this contact zone (Sutton 1938; Sibley and Short 1964; Sutton 1968; Rising 1970) led to the lumping of *I. galbula* and *I. bullockii* (including *I. abeillei*) into a single species, the northern oriole (AOU 1973). Later studies found that the *I*. *galbula–I. bullockii* hybrid zone was stable (Rohwer and Manning 1990; Rohwer and Johnson 1992; Rising 1996; Allen 2002), and full species status was restored for all three members of the group (AOU 1995). Hybridization has never been firmly documented between *I. abeillei* and the other two members of the group, but there is some evidence suggesting hybridization with *I. bullockii* where their ranges abut in northern Durango, Mexico (Miller 1906; but see Rising 1973). We are unaware of any documented instances of hybridization between members of the northern oriole group and other oriole species.

Despite the extensive research on the northern oriole group, we have limited knowledge about the extent of nuclear gene flow within this species complex. Allen (2002) examined clinal variation of phenotypic and molecular markers across the *I. galbula–I. bullockii* hybrid zone in Kansas, and found that neither male plumage traits nor mtDNA haplotypes introgressed beyond the zone of contact. These findings were recently corroborated by Carling et al. (2011), who estimated the mitochondrial cline to be 328 km wide. In contrast, of 152 neutral AFLP markers examined by Allen (2002), there were no fixed differences between the two species, and only ten AFLP bands differed more than 40% in frequency across the hybrid zone. Allen (2002) interpreted this apparent lack of differentiation in AFLP markers between *I. galbula* and *I. bullockii* as evidence of unhampered nuclear introgression since the two species came into secondary contact. However, such extensive allele sharing among closely related species could also result from the slow sorting of retained ancestral polymorphism (Moore 1995; Hudson and Turelli 2003).

The goals of this study were to (1) estimate levels of introgression among all three species in the northern oriole group while accounting for their recent shared ancestry using a three-population IMa2 model and (2) examine the effects of genetic exchange with unsampled taxa in two-population analyses.

## Material and Methods

### Taxonomic sampling

We sought a broad representation from throughout the ranges of all three species whilst avoiding known areas of sympatry (see Appendix 1 for sampling locations). Our sampling scheme provided a total of 21 to 24 individuals of each of the three species, *I. abeillei*, *I. bullockii* and *I. galbula*. In addition, a single individual of western meadowlark (*Sturnella neglecta*) was included as an outgroup taxon.

### Multilocus nuclear data

This study included sequence data from eight intron-spanning loci that map to six autosomes and the Z sex chromosome in the zebra finch (*Taeniopygia guttata*) genome (see Table 1for details). The two Z-linked loci are well separated on the zebra finch Z chromosome (>7 Mb apart) and were therefore treated as independent (Backström et al. 2006, 2010). All eight loci were amplified and sequenced following procedures described in previous phylogenetic studies on *Icterus* (Jacobsen et al. 2010; Jacobsen and Omland 2011). Resulting DNA chromatograms were aligned and edited in SEQUENCHER 4.7 (Gene Codes Corp., Ann Arbor, MI). Edited sequences for all loci were imported into MEGA5 (Tamura et al. 2011) and aligned using MUSCLE (Edgar 2004) with final manual adjustments. The haplotypes of individuals heterozygous at more than one position at a given locus were determined using the software PHASE v.2.1.1 (Stephens et al. 2001; Stephens and Scheet 2005), with the following parameter settings: burn-in = 1000, number of iterations = 10,000, and thinning interval = 1. Each analysis was repeated 10 times using different random starting seed and the run that received the highest log-likelihood was used to infer the haplotypes. To avoid systematic bias in downstream coalescent analyses, all haplotypes resolved at a probability of 0.5 or higher were included in the study (Garrick et al. 2010). An ambiguously aligned single-nucleotide repeat (poly-A) region within locus *FGB4* was excluded from the alignment prior to analyses. Linkage analysis of the two Z-linked loci (*ALDOB5* and *BRM15*) using HAPLOVIEW v.4.2 (Barrett et al. 2005) revealed no evidence of LD among polymorphic sites (results not shown). We tested for intralocus recombination using the difference in sums of squares (DSS) test in TOPALi v.2.5 (Milne et al. 2004), with 500 bootstrap replicates to determine statistical significance. A significant DSS peak was detected at locus *β-ACT2*, and only the largest non-recombinant sequence block (558 bp) was retained for downstream analysis. Accession numbers for sequences obtained from previous studies on this group are listed in Appendix 2. All new sequences collected in this study have been deposited in GenBank under accession numbers JX403068-JX403342 and JX412950-JX412955.

**Table 1:** Locus information. Chromosome: intron location determined based on BLAST searches of zebra finch reference genome assembly, with start coordinates for Z-linked loci provided in parentheses; Length: size (in base pairs) of largest non-recombinant alignment segment; *N*: number of non-recombinant alleles included in the study

Locus^1^	Chromosome	Length	*N*			Primer source

			*I. abeillei*	*I. bullockii*	*I. galbula*	
*β-ACT2*	22	558	24	26	24	(Waltari and Edwards 2002)
*α-ENO8*	21	252	46	42	46	(Kondo et al. 2008)
*FGB4*	4	575	24	30	28	(Barker 2004)
*GAPDH11*	1	327	36	30	30	(Primmer et al. 2002)
*RDP2*	12	298	36	28	26	(Waltari and Edwards 2002)
*TGFβ5*	3	575	30	32	28	(Bureš et al. 2002)
*ALDOB5*	Z (400,071)	253	36	35	24	(Kondo et al. 2008)
*BRM15*	Z (7,528,458)	362	40	35	36	(Borge et al. 2005)

1 Full locus information is as follows: Beta-actin gene, intron 2 (*β-ACT2*); alpha-enolase gene, intron 8 (*α-ENO8*); fibrinogen B beta polypeptide gene, intron 4 (*FGB4*); glyceraldehyde-3-phosphate dehydrogenase gene, intron 11 (*GAPDH11*); rhodopsin gene, intron 2 (*RDP2*); and transforming growth factor beta-2, intron 5 (*TGFβ5*); aldolase-B fructose-biphosphate intron 5 (*ALDOB5*); Brahma protein gene, intron 15 (*BRM15*).

Three common measures of nucleotide polymorphism were calculated using ARLEQUIN v.3.11 (Excoffier and Lischer 2010): number of haplotypes (h), haplotype diversity (Hd), and the average number of nucleotide differences per site between two sequences (π). Haplotype networks illustrating the relationships among inferred alleles were constructed for each locus using the median-joining algorithm in NETWORK v.4.5.1.0 (Bandelt et al. 1999, http://www.fluxus-engineering.com). The networks were edited using NETWORK PUBLISHER v.1.1.0.7 (http://www.fluxus-engineering.com).

Tests for selection were conducted using a multilocus HKA test (Hudson et al. 1987) implemented in the HKA program available at http://lifesci.rutgers.edu/∼heylab/HeylabSoftware.htm. We chose western meadowlark (*Sturnella neglecta*) as outgroup for this test, the most distant relative within the family Icteridae available for comparison across the eight loci examined in this study. Kimura 2P distances between the three northern oriole species and *S. neglecta* were calculated using DNASP v.5.10.0.1(Librado and Rozas 2009). Statistical significance of the HKA test was determined through 10,000 coalescent simulations based on the number of sampled alleles and observed levels of polymorphism and divergence across loci. These simulations also allowed us to conduct a multilocus test of Tajima's *D* statistic (Tajima 1989). Fu's (1997) *F*s and Ramos-Onsins and Rozas (2002)*R*_2_ neutrality statistics were also calculated using DnaSP. Confidence intervals and statistical significance of test values were calculated using 10,000 random permutations and coalescent simulations.

### Multilocus coalescent analyses

We examined patterns of gene flow among all three members of the northern oriole group using the Bayesian IM model implemented in the program IMa2 (Hey 2010b), which assumes random mating, constant population sizes, selective neutrality, free recombination among loci and no recombination within loci (Hey and Nielsen 2004, 2007; Hey 2010b).

By default, all model parameters estimated by IMa2 are scaled to the population mutation rate. The parameter estimates were converted into demographic units (i.e., effective population sizes in number of individuals and divergence times in years) using a neutral mutation rate of 1.35*10^−9^ substitutions per site per year for autosomal loci (Ellegren 2007) and 1.45*10^−9^ substitutions per site per year for Z-linked loci (Axelsson et al. 2004; Ellegren 2007). To reflect the different modes of inheritance, autosomal (1.0) and Z-linked (0.75) loci were assigned respective inheritance scalars in the IMa2 input file. Population sizes were converted into effective number of individuals using a generation time of 1.7 years, the average age at reproduction documented for multiple passerine lineages (Sæther et al. 2005). The actual generation time in orioles may well be slightly longer, as most oriole species exhibit delayed plumage maturation, which could delay the onset of reproduction (for males in particular) (e.g., Flood 1984; Rohwer and Manning 1990; Butcher 1991; Richardson and Burke 1999). Effective population sizes reported in this study are therefore likely conservative estimates of the actual effective population sizes. The HKY mutation model was specified for all loci.

### Three-population IMa2 analysis

First, we estimated levels of gene flow among all three species within the northern oriole group simultaneously in a three-population IMa2 analysis. We conducted separate analyses with two alternative population trees: (1) the nuclear species tree inferred by our recent species tree analysis of *Icterus* (Jacobsen and Omland 2011): (*I. galbula*, (*I. abeillei*, *I. bullockii*)) and (2) the mtDNA gene tree inferred by Omland et al. (1999): (*I. bullockii*, (*I. abeillei*, *I. galbula*)).

Short preliminary runs were conducted to determine appropriate upper bounds of the demographic parameters. Subsequently, two identical long runs of 3–5*10^6^ MCMC steps with a burnin period of 1*10^6^ steps were started with different starting seeds and run until minimum effective sample sizes (ESS) of 200 were achieved. We assessed convergence by monitoring the mixing properties of the MCMC during each run and by ensuring that similar parameter estimates were obtained from the two independent runs. Acceptable chain mixing (i.e., absence of long-term trends in plots of L[P] and *t* over the course of each run) and low autocorrelations (<0.1) were achieved by running 100 chains under a geometric heating scheme (with heating parameters set to a = 0.975 and b = 0.75). Population-specific parameter priors were provided in a separate file. We used exponential priors on migration rates (option–j7), and determined appropriate prior distribution means based on the peak posterior parameter estimates from preliminary runs.

### Two-population IMa2 analyses

To assess the effects of violating the crucial assumption of no allelic contributions from other source (i.e., “ghost”) populations, we also conducted three separate pairwise IMa2 analyses of the three northern oriole species. Run settings were kept unchanged, except for slight adjustment of parameter priors where posterior density distributions exceeded the upper bounds determined by preliminary runs. Each MCMC (M) mode analysis was replicated using different random seeds to ensure convergence onto similar posterior distributions, using the same criteria for assessing convergence as described above. Finally, we conducted nested model testing by running IMa2 in “Load genealogies” (L) mode to specifically compare the fit of reduced models that do not allow for post divergence gene flow to the full IMa2 model (i.e., including all six parameters Θ_1_, Θ_2_, Θ_a_, m_1_, m_2_, t). This was performed by sampling 2.5*10^5^ genealogies evenly from the two MCMC runs performed on each population pair and compared the log-likelihood scores of all possible nested models provided in a separate file in the IMa2 distribution package.

## Results

### Polymorphism and divergence

The three species within the northern oriole group were polymorphic at all loci used in this study, except for *I. abeillei* that was monomorphic at *ALDOB5*. Sample sizes and data for individual loci are summarized in Table 1. The three measures of sequence polymorphism were all lower in *I. abeillei* than in *I. bullockii* and *I. galbula* (Table 2), but not significantly so (single factor ANOVA: h: *F*_2,21_ = 0.656, *P* = 0.529; Hd: *F*_2,21_ = 0.585, *P* = 0.566; π: *F*_2,21_ = 0.943, *P* = 0.405; respectively). The differences in mean genetic diversity between *I. abeillei* and the two other members of the group are mainly driven by the lack of sequence variation at the sex-linked locus *ALDOB5* (Table 2).

**Table 2:** Locus-specific estimates of number of haplotypes (h), haplotype diversity (Hd), and Jukes & Cantor nucleotide diversity (π)

	*I. abeillei*			*I. bullockii*			*I. galbula*		
			
Locus	h	Hd	*π*	h	Hd	π	h	Hd	π
*β-ACT2*	5	0.656	0.00147	4	0.397	0.00094	4	0.239	0.00060
*α-ENO8*	7	0.283	0.00136	9	0.490	0.00224	9	0.481	0.00234
*FGB4*	10	0.786	0.00300	20	0.956	0.00741	15	0.942	0.00603
*GAPDH11*	8	0.830	0.00472	8	0.823	0.00492	14	0.883	0.00590
*RDP2*	7	0.711	0.00385	7	0.804	0.00438	3	0.578	0.00216
*TGFβ5*	3	0.393	0.00087	6	0.585	0.00213	14	0.923	0.00430
*ALDOB5*	1	0	0	4	0.395	0.00217	3	0.507	0.00372
*BRM15*	3	0.145	0.00068	4	0.217	0.00062	2	0.413	0.00114

**Figure 3:**
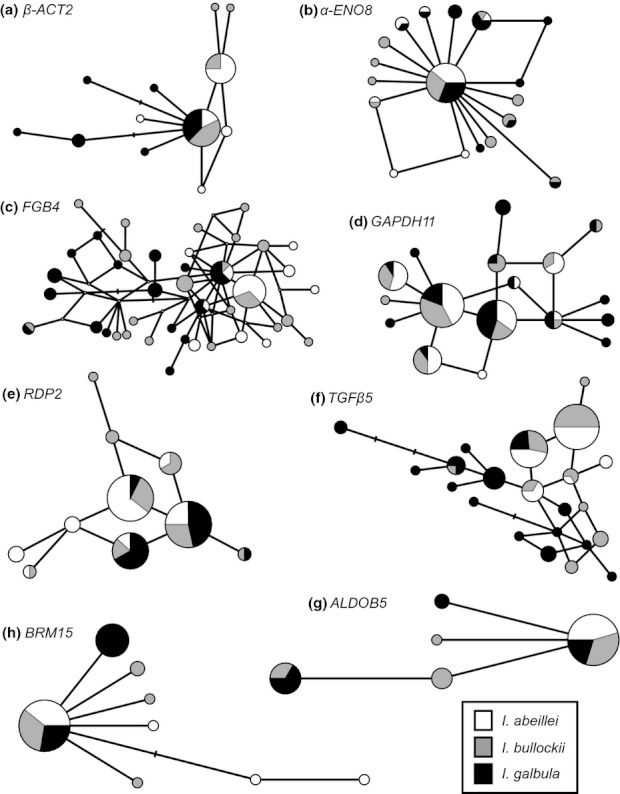
Haplotype networks of eight nuclear intron loci (6 autosomal + 2 Z-linked) revealing extensive haplotype sharing among the three northern orioles: *Icterus abeillei* (white), *I. bullockii* (gray), and *I. galbula* (black). Each circle corresponds to a unique haplotype and the size of each circle reflect the relative total sample size of each haplotype. Small open circles in (c) indicate unsampled/extinct haplotypes, and perpendicular bars along lines connecting haplotypes indicate additional mutations distinguishing neighboring haplotypes.

The haplotype networks revealed extensive allele sharing and few fixed differences among the three northern oriole species (Fig. 3). Some loci were characterized by a distinct star-shaped topology (e.g., *α-ENO8*, *ALDOB5* and *BRM15*), with a common central (and presumed ancestral) haplotype surrounded by several rare, derived haplotypes one or two mutational steps away from the ancestral haplotype (Fig. 3b,g,h).

The three species showed varying degrees of genetic differentiation across loci, but generally exhibited extensive haplotype sharing across loci (Table 3, also see Fig. 3). Strongest differentiation was observed between the highly disjunct *I. abeillei* and *I. galbula*, with highly significant F_ST_ values at six of the eight loci examined. The two widely hybridizing *I. bullockii* and *I. galbula* were strongly differentiated at only half (4/8) of the loci examined. The two sister species, *I. abeillei* and *I. bullockii*, were only weakly differentiated and only two loci (*FGB4* and *ALDO5*) remained significant after Bonferroni corrections (Table 3).

**Table 3:** Population differentiation among three northern oriole populations, *Icterus abeillei*, *I. bullockii*, and *I. galbula*, estimated based on pairwise genetic distances between populations. Significant F_ST_ values are indicated at the 0.05 > *P* > 0.01 (*) and *P* < 0.01 (**) level. Bolded values are significant at the Bonferroni-adjusted α-level of 0.00625

F_ST_			

Locus	*I. abeillei–I. bullockii*	*I. abeillei–I. galbula*	*I. bullockii–I. galbula*
*β-ACT2*	0.151**	0.372**	0.096*
*α-ENO8*	0.010	0.019*	0.019*
*FGB4*	0.102**	0.383**	0.173**
*GAPDH11*	0	0.064*	0.052*
*RDP2*	0.069*	0.289**	0.177**
*TGFβ5*	0.065*	0.400**	0.289**
*ALDOB5*	0.131**	0.241**	0.017
*BRM15*	0.018	0.186**	0.186**

The multilocus HKA test indicated no significant departures from neutrality in our dataset. The multilocus Tajima's *D* test was significant only for *I. bullockii*, a result that is mainly driven by significantly more negative than expected *D* values at loci *α-ENO8* and *BRM15* (Table 4). *Fs* values were negative at the majority of loci, indicative of recent population growth, but this statistic deviated only significantly from neutral expectations at less than half the loci following Bonferroni corrections (Table 4). The *R*_2_ test was only significant at a single locus *α-ENO8* (Table 4).

**Table 4:** Locus-specific tests for deviations from selective neutrality and constant population sizes in *Icterus abeillei* (a), *I. bullockii* (b), and *I. galbula* (g). Significance of observed values is indicated at the 0.05 > *P* > 0.01 (*) and *P* < 0.01 (**) level. Tests were inapplicable for *I. abeillei* at *ALDOB5* due to lack of sequence variation. Bolded values remained significant after Bonferroni corrections for multiple testing (adjusted α-level of 0.00625)

	Tajima's *D*			Fu's *F*_s_			Ramos-Onsins & Rozas *R*_2_		
			
Locus	a	b	g	a	b	g	a	b	g
*β-ACT2*	0.048	−0.821	−1.884**	−1.406	−1.227	−1.398	0.14	0.108	0.118
*α-ENO8*	−1.725*	−1.97**	−1.708*	−6.561**	−7.748**	−7.315**	0.048	0.043**	0.05*
*FGB4*	−1.412	−0.24	0.711	−4.64**	−11.431**	−6.177**	0.085	0.118	0.152
*GAPDH11*	0.712	0.152	−0.993	−1.889	−2.089	−8.774**	0.154	0.131	0.078
*RDP2*	−0.136	0.019	0.474	−1.978	−1.962	0.464	0.114	0.126	0.16
*TGFβ5*	0.024	−0.047	−0.144	0.058	−0.973	−7.268**	0.125	0.13	0.128
*ALDOB5*	–	−0.559	0.434	–	−0.88	1.258	–	0.099	0.157
*BRM15*	−1.423	−1.559*	1.047	−1.112	-2.963**	1.369	0.061	0.08	0.206

### Three-population IMa2 analysis

**Figure 4:**
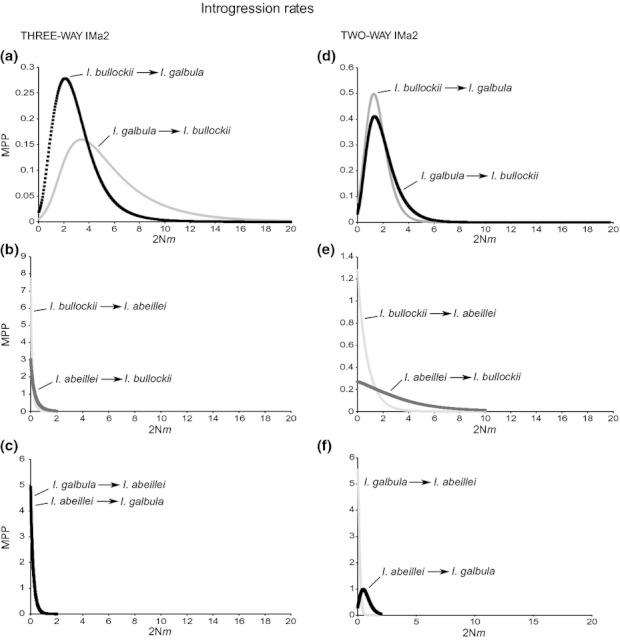
Posterior probability plots of bi-directional migration rates (given in effective number of gene copies - 2N*m*) estimated between *Icterus bullockii* and *I. galbula* (a,d), between *I. abeillei* and *I. bullockii* (b,e), and between *I. abeillei* and *I. galbula* (c,f) based on a combined three-population analysis and three separate pairwise analyses in IMa2.

The inclusion of all three members of this species complex in a single IMa2 analysis revealed some interesting patterns of gene introgression (Fig. 4). The two competing population trees (nDNA vs. mtDNA) yielded similar parameter estimates (with one important exception discussed below). We therefore only report the results based on the nuclear population tree (*I. galbula*, (*I. abeillei, I. bullockii*)).

First, we found clear evidence of substantial introgression across the hybrid zone between *I. galbula* and *I. bullockii*, (2N*m*_*I. galbula* > *I. bullockii*_ = 3.388, 95% HPD = 0.41–12.30, vs. 2N*m*_*I. bullockii* > *I. galbula*_ = 2.125, 95% HPD = 0.193–6.504, Fig. 4a). Interestingly, the gene flow appears to be slightly skewed in the direction of *I. bullockii*. In contrast, we did not detect any gene flow between the sister species *I. bullockii* (Fig. 4b). The gene flow estimates virtually peaked at zero (2N*m* = 0.001 in both directions) and the 95% HPD intervals ranged from 0 to 1.148 and 0 to 0.495 in the direction of *I. bullockii* and *I. abeillei*, respectively. Similarly, the three-population analysis did not detect any gene flow between the highly allopatric *I. galbula* and *I. abeillei* (Fig. 4c), with gene flow estimates peaking near zero and 95% HPD intervals ranging from 0 to 0.631 and 0 to 0.719 in the direction of *I. galbula* and *I. abeillei*, respectively. There was no evidence of historical gene flow between the *I. galbula* lineage and the ancestral *I. abeillei/I. bullockii* lineage.

**Figure 5:**
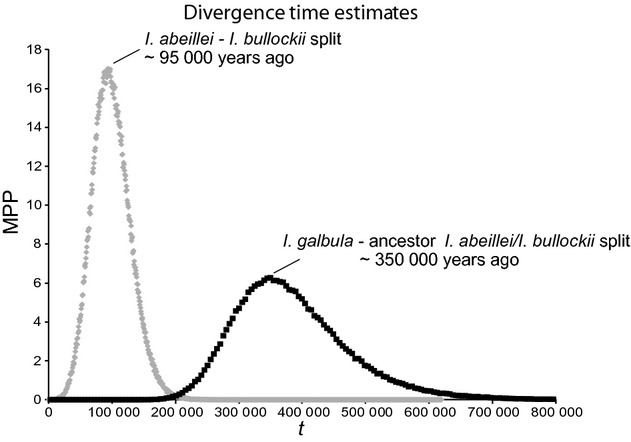
Posterior probability plot of divergence (splitting) times within the northern oriole group. According to IMa2, the initial split between *Icterus galbula* and the ancestor of *I. abeillei* and *I. bullockii* occurred roughly 350,000 years ago, whereas the more recent split between *I. abeillei* and *I. bullockii* occurred approximately 95,000 years ago.

**Figure 6:**
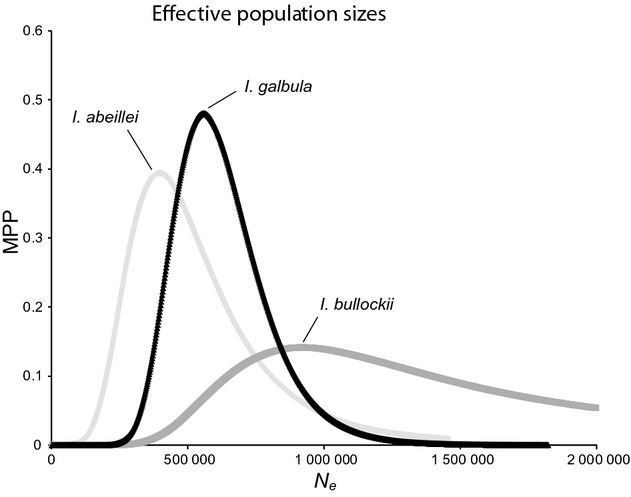
Posterior probability plot of effective population sizes (*N*_e_) of extant populations of *I. abeillei*, *I. bullockii*, and *I. galbula*. Peak estimates of *N*_e_ are very similar for all three populations, with highly overlapping confidence intervals (95% HPDs).

IMa2 provided very concise estimates of the timing of divergence events within the group. The analysis based on the nuclear species tree suggested that the initial divergence between the *I. galbula* lineage and the ancestor of *I. abeillei* and *I. bullockii* appears to have occurred approximately 350,000 years ago (95% HPD = 215,838–568,125), whereas *I. abeillei* and *I. bullockii* split much more recently approximately 95,000 years ago (41,865–159,087, Fig. 5a). In contrast, the analysis based on the mtDNA population tree (*I. bullockii*, (*I. abeillei*, *I. galbula*)) resulted in perfectly overlapping estimates of the two splitting events around 215–250,000 years ago, with nearly perfectly overlapping HPD intervals (Fig. 5b). Effective population sizes (*N*_*e*_) were approximately 398,000 black-backed orioles, 557,000 Baltimore orioles, and 921,000 Bullock's orioles (Fig. 6).

### Two-population IMa2 analyses

A summary of the three separate two-population analyses is presented in Table 3. Estimates of gene flow were mostly consistent between the pairwise two-population analyses and the three-population analysis. However, the two-population analysis of *I. abeillei* and *I. galbula* suggested a small but significant amount of introgression from *I. abeillei* into *I. galbula* (2N*m*_*I. abeillei > I. galbula*_ = 0.477 (Fig. 4f). Although the 95% HPD interval included zero (see Table 5), nested model testing conducted in IMa2 indicated that a reduced model with zero migration was a significantly worse fit to the data than the full model allowing for migration (results not shown). Furthermore, the two-population analysis of *I. galbula* and *I. bullockii* indicated lower but equal rates of introgression in both directions (2N*m*_*I. bullockii* > *I. galbula*_ = 1.331 and 2N*m*_*I. galbula* > *I. bullocki*_ = 1.275, see Fig. 4d).

**Table 5:** Summary of pairwise two-population IMa2 analyses of black-backed oriole (*Icterus abeillei*), Bullock's oriole (*I. bullockii*), and Baltimore oriole (*I. galbula*). Given are highest posterior parameter estimate (HiPt) and lower (HPD95Lo) and upper (HPD95Hi) bounds of 95%HPD intervals for the effective population size of population 1 (N_1_), population 2 (N_2_), and ancestral population (N_a_), splitting time (t), and population migration rate from population 2 into population 1 (2N*m*1 > 2) and from population 1 into population 2 (2N*m*2 > 1) forward in time. Population sizes are given in 1000 individuals and time since divergence in 1000 years

	N_1_	N_2_	N_a_	t	2N*m*1 > 2	2N*m*2 > 1
*I. abeillei* (1) vs. *I. galbula* (2)						
HiPt	328	951	169	411	0.001	0.477
HPD95Lo	200	532	66	216	0	0
HPD95Hi	527	1,937	332	750	0.351	1.486
*I. abeillei* (1) vs. *I. bullockii* (2)						
HiPt	517	1,198	225	163	0.005	0.025
HPD95Lo	252	528	118	79	0	0
HPD95Hi	1,184	4,732	373	299	2.404	7.461
*I. bullockii* (1) vs. *I. galbula* (2)						
HiPt	595	688	163	424	1.275	1.331
HPD95Lo	350	397	62	245	0	0.069
HPD95Hi	1,005	1,215	313	749	3.206	4.25

The divergence time estimates from the pairwise two-population analyses were comparable to those produced by the three-population IMa2 analysis, except that both divergence events (*I. galbula*–*I. abeillei/I. bullockii* split, and *I. abeillei*–*I. bullockii* split) were estimated to have occurred roughly 70,000 years earlier than that indicated by the three-population analysis (see previous section, Fig. 5). In agreement with the nuclear species tree of this group (Jacobsen and Omland 2011), the most recent divergence in this group between *I. abeillei* and *I. bullockii* was estimated at approximately 163,000 years ago, well within the 95% HPD interval of the three-population estimate (see previous section). Similarly, the older split between the *I. galbula* lineage and the sister taxa *I. abeillei* and *I. bullockii* were estimated at 411,000 and 424,000 years ago, respectively.

Effective population size (*N*_*e*_) estimates from the pairwise two-population analyses were also roughly similar to those estimated by the three-population analysis. The peak estimates were slightly higher in the two-population analyses, but the 95% HPD intervals overlap greatly with the estimates from the three-population analysis. The most striking deviations in population size estimates arose when the non-sister species, *I. galbula* and *I. abeillei*, were analyzed separately, which resulted in overestimation of *N*_*I. galbula*_ and a slight underestimation of *N*_*I. abeillei*_ (Table 5). Similarly, *N*_*I. bullockii*_ was substantially smaller when paired with the non-sister *I. galbula* than when correctly paired with its sister species *I. abeillei* (Table 5, Fig. 6).

## Discussion

The recent development of a multispecies coalescent framework for the study of divergence and gene flow among three or more taxa simultaneously in the program IMa2 (Hey 2010b) has proven useful for understanding the population dynamics of complex taxonomic groups consisting of several recently diverged species (e.g., Hey 2010a; Illera et al. 2011; Schoville et al. 2011; So et al. 2011; Li et al. 2012). In this study, we were able to estimate levels of nuclear introgression between allopatric populations of the three species in the northern oriole group while also accounting for their recent shared ancestry.

The hybrid zone between the non-sister species, *I. galbula* and *I. bullockii*, represents perhaps one of the most blatant case examples for the need of a multipopulation framework to disentangle the demographic history of larger groups of taxa. The reproductive barriers that so effectively prevent mtDNA and phenotypes of both *I. galbula* and *I. bullockii* from introgressing beyond the stable hybrid zone appear to be “leaky”, allowing near-neutral nuclear alleles to introgress and successfully spread in a heterospecific genetic background without erasing the diagnostic species differences. Such a substantial rate of introgression evident in populations several hundred miles away from the nearest known areas of sympatry has major implications for our understanding of the population dynamics of all three species within the group. Limited mtDNA introgression (Allen 2002; Carling et al. 2011) but extensive nDNA introgression (Allen 2002; this study) follows the predictions of Haldane's rule (Haldane 1922), wherein reduced female hybrid fitness prevents maternally inherited mtDNA, but not bi-parentally inherited nDNA from introgressing (Brookfield 1993; Turelli and Orr 1995; Orr 1997). Sex-biased (i.e., male driven) gene flow due to female heterogamety and the effects of Haldane's rule have increasingly been documented in a wide range of avian groups (e.g., Borge et al. 2005; Carling and Brumfield 2008; Carling et al. 2010; Storchová et al. 2010; Backström and Väli 2011).

A question of interest to researchers studying divergence and gene flow is to determine the timing of migration events between diverging populations. If possible, knowing when gene flow occurred in the speciation process would be of great importance to distinguish between an allopatric speciation model with recent secondary contact following range expansion (gene flow late) and a parapatric speciation model where divergence occurred in the presence of gene flow with subsequent decrease, then cessation of gene flow once reproductive barriers evolved. However, it is now clear that IMa2 and similar methods are unable to distinguish between these different modes of speciation (Sousa et al. 2011; Strasburg and Rieseberg 2011). We therefore cannot infer with confidence whether the currently hybridizing *I. galbula* and *I. bullockii* diverged in allopatry and only recently came back into secondary contact following postglacial range expansion, or if they diverged in parapatry while still exchanging genes throughout the early stages of divergence. Nonetheless, the fact that these two orioles are nearly 5% divergent in mtDNA (Omland et al. 1999) indicates that they have been evolving independently for an extended period of time, consistent with allopatric speciation followed by one or more episodes of gene flow during the last glacial cycles of the Pleistocene epoch. Pliocene or Pleistocene divergence followed by postglacial expansion has been proposed to explain the current distribution of many parapatric species found in North America and Eurasia (Klicka and Zink 1999; Hewitt 2004; Johnson and Cicero 2004). A recent exchange of nuclear variation through introgressive hybridization during secondary contact in the late Pleistocene would explain the wide gap between the mtDNA and nDNA divergence time estimates for these two species, even though the TMRCA for a single locus (e.g., mtDNA) will necessarily always predate the divergence between two species (Edwards and Beerli 2000).

In contrast, the IMa2 analyses revealed no evidence of introgression between *I. abeillei* and *I. bullockii*. The region of parapatry in Durango, Mexico where hybridization between these two species is alleged to have taken place was unfortunately not represented in this study. It is therefore possible that some hybridization could occur within this region, but restricted enough to prevent widespread introgression into the core ranges of either species. Thus, the ubiquitous nuclear haplotype sharing observed between these sister species most likely represents ancestral polymorphism retained in both descendent lineages since their recent split in the late Pleistocene (Hudson and Turelli 2003)

The low rate of unidirectional introgression from *I. abeillei* into *I. galbula* that was indicated by the two-population IMa2 analysis is somewhat puzzling, given that no evidence of gene flow between these two species was revealed in the three-population analysis. Previous studies on these two species using IM based on mtDNA and two nuclear introns also found no evidence of gene flow between these two mitochondrial sister taxa (Kondo et al. 2004, 2008). The fact that they are reciprocally monophyletic at the mtDNA level separated by a single fixed substitution also strongly suggests that there is no recent or ongoing mitochondrial gene flow between these highly allopatric species (Kondo et al. 2008).

**Figure 7:**
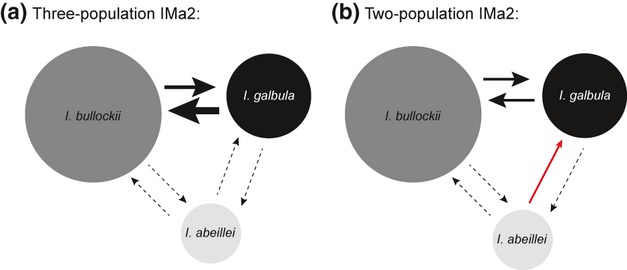
Patterns of gene flow among the three species in the northern oriole group estimated using IMa2. Circle sizes reflect relative *N*_e_ estimates. Relative rates of gene introgression are indicated by the width of solid arrows. Dashed arrows indicate little or no introgression. (a) The full three-population analysis inferred gene flow only between the hybridizing *Icterus galbula* and *I. bullockii*, whereas (b) two-population analyses also indicated a low level of introgression (highlighted in red) from *I. abeillei* into *I. galbula*. The introgression between these highly allopatric species is best ascribed to ancestral polymorphisms shared between *I. abeillei* and *I. bullockii* introduced to *I. galbula* across the *I. galbula*–*I. bullockii* hybrid zone. This study clearly illustrates how introgression from other taxa not included in the analysis (in this case *I. bullockii*) can bias gene flow estimation if not accounted for.

Rather, we suspect that this signature of gene flow is a spurious outcome arising when violating the assumption of the IM model that no genetic exchange has occurred between the two focal populations and ghost populations (Beerli 2004; Slatkin 2005). As *I. abeillei* shares a more recent evolutionary history with *I. bullockii* than with *I. galbula* (Jacobsen et al. 2010; Jacobsen and Omland 2011), retained ancestral polymorphisms shared between *I. abeillei* and *I. bullockii* could introgress into *I. galbula* via *I. bullockii* over the extensive hybrid zone. This study revealed just how easily neutral nuclear alleles can cross this species boundary and freely introgress across the entire range of the other species. By excluding *I. bullockii* from the analysis, such shared ancestral alleles could mistakenly be inferred by IMa2 to have introgressed directly from *I. abeillei* into *I. galbula* (Fig. 7). Furthermore, the many alleles introgressing from *I. bullockii* into *I. galbula* have probably contributed to the high nucleotide diversity in *I. galbula*, causing IMa2 to overestimate the current effective population size of *I. galbula* in analyses that do not account for *I. bullockii*.

Thus, exclusion from the analysis of other taxa that have exchanged genes with either of the two focal taxa through shared ancestry or gene flow can bias estimation of population sizes and gene flow in IM, and the severity of the bias depends on how large the genetic contribution from the third taxon has been (Strasburg and Rieseberg 2010).

## Conclusions

Our study reveals the complexity of evolutionary processes (recent divergence, slow sorting of ancestral variation, and introgressive hybridization) that have contributed to produce the observed pattern of rampant allele sharing among the three species within the northern oriole group. Furthermore, our findings highlight the importance of accounting for all potentially interbreeding taxa when interpreting the divergence history of a group of taxa, and the extent to which gene flow may retard the rate of divergence at neutral loci. Thus, this study provides an interesting perspective on the broad-scale impacts of introgressive hybridization, which has important implications for everything from understanding adaptive evolution to inferring species trees. The ease with which genome-scale datasets can now be obtained will soon enable researchers to study even more complex clades with four or more taxa that may interbreed.

## Appendix

**Appendix 1:** Taxonomic sampling

Species	Museum^a^	ID	Sex	Collection Locality^b^
*Icterus abeillei*	MZFC	2339	M	Mexico, Morelos, Carretera
*Icterus abeillei*	MZFC	5381	M	Mexico, Guerrero, Apaxtla
*Icterus abeillei*	MZFC	3468	M	Mexico, Guanajuato, San Pedro
*Icterus abeillei*	MZFC	KEO27	F	Mexico: Guanajuato, Salvatierra
*Icterus abeillei*	MZFC	KEO28	M	Mexico: Guanajuato, Salvatierra
*Icterus abeillei*	MZFC	KEO29	M	Mexico: Guanajuato, Salvatierra
*Icterus abeillei*	MZFC	1087	M	Mexico, Guanajuato, Yuriria
*Icterus abeillei*	MZFC	5441	M	Mexico, Guanajuto, Yuriria
*Icterus abeillei*	MZFC	KEO30	M	Mexico: Guanajuato, Yuriria
*Icterus abeillei*	MZFC	KEO31	M	Mexico: Guanajuato, Yuriria
*Icterus abeillei*	MZFC	3467	M	Mexico, Jalisco, Laguna de Chapala
*Icterus abeillei*	MZFC	7966	M	Mexico: Jalisco
*Icterus abeillei*	MZFC	10733	M	Mexico: Jalisco
*Icterus abeillei*	MZFC	11483	M	Mexico: Jalisco
*Icterus abeillei*	MZFC	KEO32	M	Mexico: Jalisco, Chapala
*Icterus abeillei*	MZFC	KEO33	M	Mexico: Jalisco, Chapala
*Icterus abeillei*	MZFC	KEO34	M	Mexico: Jalisco, Chapala
*Icterus abeillei*	MZFC	KEO35	F	Mexico: Jalisco, Chapala
*Icterus abeillei*	MZFC	KEO37	F	Mexico: Jalisco, Chapala
*Icterus abeillei*	MZFC	KEO45	M	Mexico: Michoacán, Briseñas
*Icterus abeillei*	MZFC	KEO46	M	Mexico: Michoacán, Briseñas
*Icterus abeillei*	MZFC	KEO48	F	Mexico: Michoacán, Briseñas
*Icterus abeillei*	MZFC	KEO49	M	Mexico: Michoacán, Briseñas
*Icterus bullockii*	MVZ	176515	M	USA: NM, Mora Co.
*Icterus bullockii*	LSUMNS	3874	F	USA: CA, Inyo Co.
*Icterus bullockii*	LSUMNS	3875	F	USA: CA, Inyo Co.
*Icterus bullockii*	LSUMNS	3876	M	USA: CA, Inyo Co.
*Icterus bullockii*	LSUMNS	3877	F	USA: CA, Inyo Co.
*Icterus bullockii*	LSUMNS	3878	M	USA: CA, Inyo Co.
*Icterus bullockii*	LSUMNS	3879	M	USA: CA, Inyo Co.
*Icterus bullockii*	LSUMNS	3880	M	USA: CA, Inyo Co.
*Icterus bullockii*	FMNH	330029	M	USA: CA, San Bernardino Co.
*Icterus bullockii*	FMNH	341933	M	USA: CA, Imperial Co.
*Icterus bullockii*	FMNH	341934	M	USA: CA, Monterrey Co.
*Icterus bullockii*	FMNH	341937	M	USA: CA, Monterrey Co.
*Icterus bullockii*	FMNH	341938	M	USA: CA, Monterrey Co.
*Icterus bullockii*	UWBM	55942	M	USA: WA, Grant Co.
*Icterus bullockii*	UWBM	55951	F	USA: WA, Douglas Co.
*Icterus bullockii*	UWBM	55975	M	USA: WA, Douglas Co.
*Icterus bullockii*	UWBM	55976	F	USA: WA, Douglas Co.
*Icterus bullockii*	UWBM	55978	F	USA: WA, Douglas Co.
*Icterus bullockii*	UWBM	59055	F	USA: WA, Asotin Co.
*Icterus bullockii*	UWBM	59056	M	USA: WA, Asotin Co.
*Icterus bullockii*	UWBM	59058	M	USA: WA, Asotin Co.
*Icterus galbula*	LSUMNS	131146	M	USA: LA, Cameron Co.
*Icterus galbula*	ANSP	10126	M	USA: PA, Bucks Co.
*Icterus galbula*	ANSP	10134	M	USA: PA, Bucks Co.
*Icterus galbula*	ANSP	10148	F	USA: PA, Bucks Co.
*Icterus galbula*	USFWS	17860	M	USA: MD, Baltimore Co.
*Icterus galbula*	USFWS	17861	M	USA: MD, Baltimore Co.
*Icterus galbula*	USFWS	17862	M	USA: MD, Baltimore Co.
*Icterus galbula*	USFWS	17864	M	USA: MD, Baltimore Co.
*Icterus galbula*	USFWS	17866	M	USA: MD, Baltimore Co.
*Icterus galbula*	USFWS	17867	M	USA: MD, Baltimore Co.
*Icterus galbula*	USFWS	17869	M	USA: MD, Baltimore Co.
*Icterus galbula*	USFWS	17870	M	USA: MD, Baltimore Co.
*Icterus galbula*	UWBM	67201	M	USA: MD, Prince Georges Co.
*Icterus galbula*	BMNH	42547	M	USA: MN, Becker Co.
*Icterus galbula*	BMNH	X7630	M	USA: MN, Hennepin Co.
*Icterus galbula*	BMNH	X7763	F	USA: MN, Chisago Co.
*Icterus galbula*	BMNH	X7799	M	USA: MN, Chisago Co.
*Icterus galbula*	FMNH	350604	M	USA: IL, Cook Co.
*Icterus galbula*	FMNH	395866	F	USA: IL, Cook Co.
*Icterus galbula*	HLG	LG03	M	Canada: Manitoba
*Icterus galbula*	HLG	LG04	M	Canada: Manitoba
*Icterus galbula*	HLG	LG05	M	Canada: Manitoba
*Icterus galbula*	HLG	LG06	F	Canada: Manitoba
*Sturnella neglecta*	NMNH	586115		

a Museums are abbreviated as follows: ANSP = Academy of Natural Sciences, Philadelphia; BMNH = J. F. Bell Museum of Natural History, University of Minnesota; FMNH = Field Museum of Natural History; HLG = H. Lisle Gibbs, blood samples; LSUMNS = Louisiana State University, Museum of Natural Science; MVZ = Museum of Vertebrate Zoology, University of California, Berkeley; MZFC = Museo de Zoología, Facultad de Ciencias, Universidad Nacional Autónoma de México; NMNH = National Museum of Natural History; UWBM = University of Washington, Burke Museum and USFWS = United States Fish and Wildlife Service band numbers.

b San Pedro is the short form of San Pedro de Los Naranjos, and Briseñas is the short form of Briseñas de Matamoros.

**Appendix 2:** GenBank accession numbers for DNA sequences obtained from previous studies on *Icterus* (Kondo et al. 2008; Jacobsen et al. 2010; Jacobsen and Omland 2011)

Species	GenBank Accession Nos.
*I. abeillei*	DQ898412-DQ898433, DQ898358-DQ898385, GU972843, GU972875, JN169143-JN169144, JN169230-JN169239, JN169317-JN169326, JN169404-JN169413, JN169491-JN169500, JN169578-JN169587, JN169665-JN169674
*I. bullockii*	JN169159-JN169170, JN169246-JN169257, JN169333-JN169344, JN169422-JN169431, JN169507-JN169518, JN169594-JN169605, JN169681-JN169692
*I. galbula*	DQ898316-DQ898331, DQ898344-DQ898347, DQ898356-DQ898357, DQ898386-DQ898392, DQ898407, GU972855, GU972887, JN169191- JN169196, JN169274-JN169283, JN169361-JN169370, JN169448-JN169457, JN169535-JN169544, JN169622-JN169631, JN169709- JN169718
*S. neglecta*	GU972873, GU972905
